# Omentin1 ameliorates myocardial ischemia-induced heart failure via SIRT3/FOXO3a-dependent mitochondrial dynamical homeostasis and mitophagy

**DOI:** 10.1186/s12967-022-03642-x

**Published:** 2022-10-04

**Authors:** Jingui Hu, Tao Liu, Fei Fu, Zekun Cui, Qiong Lai, Yuanyuan Zhang, Boyang Yu, Fuming Liu, Junping Kou, Fang Li

**Affiliations:** 1 Jiangsu Key Laboratory of TCM Evaluation and Translational Research, Research Center for Traceability and Standardization of TCMs, School of Traditional Chinese Pharmacy, China Pharmaceutical University, 639 Longmian Road, Nanjing, 211198 People’s Republic of China; 2 Jiangsu Province Hospital of Chinese Medicine, Affiliated Hospital of Nanjing University of Chinese Medicine, Nanjing, 210029 China

**Keywords:** Omentin1, Heart failure, Sirtuin 3, Mitochondrial dynamical homeostasis, Mitophagy, Heart-adipose crosstalk

## Abstract

**Background:**

Adipose tissue-derived adipokines are involved in various crosstalk between adipose tissue and other organs. Omentin1, a novel adipokine, exerts vital roles in the maintenance of body metabolism, insulin resistance and the like. However, the protective effect of omentin1 in myocardial ischemia (MI)-induced heart failure (HF) and its specific mechanism remains unclear and to be elucidated.

**Methods:**

The model of MI-induced HF mice and oxygen glucose deprivation (OGD)-injured cardiomyocytes were performed. Mice with overexpression of omentin1 were constructed by a fat-specific adeno-associated virus (AAV) vector system.

**Results:**

We demonstrated that circulating omentin1 level diminished in HF patients compared with healthy subjects. Furthermore, the fat-specific overexpression of omentin1 ameliorated cardiac function, cardiac hypertrophy, infarct size and cardiac pathological features, and also enhanced SIRT3/FOXO3a signaling in HF mice. Additionally, administration with AAV-omentin1 increased mitochondrial fusion and decreased mitochondrial fission in HF mice, as evidenced by up-regulated expression of Mfn2 and OPA1, and downregulation of p-Drp1(Ser616). Then, it also promoted PINK1/Parkin-dependent mitophagy. Simultaneously, treatment with recombinant omentin1 strengthened OGD-injured cardiomyocyte viability, restrained LDH release, and enhanced the mitochondrial accumulation of SIRT3 and nucleus transduction of FOXO3a. Besides, omentin1 also ameliorated unbalanced mitochondrial fusion-fission dynamics and activated mitophagy, thereby, improving the damaged mitochondria morphology and controlling mitochondrial quality in OGD-injured cardiomyocytes. Interestingly, SIRT3 played an important role in the improvement effects of omentin1 on mitochondrial function, unbalanced mitochondrial fusion-fission dynamics and mitophagy.

**Conclusion:**

Omentin1 improves MI-induced HF and myocardial injury by maintaining mitochondrial dynamical homeostasis and activating mitophagy via upregulation of SIRT3/FOXO3a signaling. This study provides evidence for further application of omentin1 in cardiovascular diseases from the perspective of crosstalk between heart and adipose tissue.

**Supplementary Information:**

The online version contains supplementary material available at 10.1186/s12967-022-03642-x.

## Introduction

Heart failure is a global medical problem and complex clinical syndrome, which is the last stage of various cardiovascular diseases, especially ischemic heart disease [1]. Due to chronic nutrient deficiency and hypoxia in the heart, dysfunctional cardiomyocytes contribute to myocardial necrosis, thereby ensuing myocardial inflammation, hypertrophy, and fibrosis, all of which act as the foundation of HF development [2]. Contemporarily, the main therapeutic drugs have limited symptomatic relief and serious adverse reactions during long-term clinical treatment, so far, the drug treatment of HF is far from sufficiency.

The increasing fatality of obesity complications such as cardiovascular diseases and the like has brought widespread attention to the research aimed at understanding the potential molecular mechanisms by which obesity contributes to the development of ischemic heart disease [3]. Adipose tissue, an endocrine organ, secreted various adipokines such as adiponectin, leptin, resistin, and the rest, which could directly affect the surrounding or remote tissues and were closely associated with the increased risk of HF [4]. Omentin, a novel circulating adipokine, is widely expressed in human omental and visceral adipose tissue. Clinical studies have found that serum omentin1 significantly decreased in patients with coronary artery disease, and could be served as a novel prognostic indicator of risk stratification in HF patients [5, 6]. Moreover, previous studies have demonstrated that omentin could attenuate myocardial ischemia reperfusion injury (MIRI) through AMPK- and Akt-dependent mechanisms [7], and it also attenuated glucocorticoid excess-induced cardiac injury through activation of GSK3β pathway [8]. Furthermore, omentin could ameliorate doxorubicin-induced H9c2 cell apoptosis [9]. Similarly, most cross-sectional studies reported that the circulating omentin1 levels were lower in patients with ischemic stroke, metabolic syndrome, or type 2 diabetes compared with subjects without cardiovascular diseases [10–12]. All of these are consistent with observations from several studies that omentin1 levels were inversely associated with multiple cardiovascular risk factors including BMI, age, waist circumference, insulin resistance, glucose levels, and lipid levels [13, 14]. In contrast, longitudinal studies have observed that higher omentin1 concentrations were connected with a higher risk of cardiovascular events in diabetes patients and with a higher risk of stroke in healthy individuals [15, 16]. However, the reasons for such discrepant findings are unclear, demonstrating that the biological roles of omentin1 in cardiovascular biology are hardly understood. Therefore, we firstly investigated the effects of omentin1 in the therapy for HF, and further clarified its potential mechanism, which could provide new strategies for the prevention and therapy of HF from crosstalk between the heart and adipose tissue.

Mitochondrial homeostasis is regulated by mitochondrial dynamics and mitophagy, which preserve organelle structure and function. Mitochondrial fission irreversibly separates damaged mitochondria, forming small sphere-shaped organelles. Conversely, Mitochondrial fusion occurs in the dynamic repair of reversible mitochondrial damage, forming functional elongated organelles [17]. Mitophagy is essential for sustaining mitochondrial homeostasis by clearing away defective mitochondria, while it may contribute to cell death if not strictly controlled [18]. HF, a bioenergetic disease, is characterized by disrupted mitochondrial homeostasis, including impaired ‘myocardial power grid’ and increased mitochondrial ROS (mitoROS) production, which is a therapeutic target for heart failure [19]. And whether adipocytokine omentin1 released in adipose tissue could exert a myocardial protective effect by acting on the heart and maintaining mitochondrial homeostasis is still worthy of further study.

SIRT3 is a NAD-dependent deacetylase in mitochondria, which regulates the mitochondrial network mainly by regulation of lysine acetylation. SIRT3 could delay the progression of heart failure through improvement of mitochondrial function including mitochondrial quality control, mitochondrial biosynthesis, oxidative phosphorylation, and fatty acid oxidation [20]. According to previous studies, SIRT3 could alleviate myocardial infarction by inhibiting AMPK-Drp1-mediated mitochondrial fission [21]. However, SIRT3 deficiency caused hyperacetylation of OPA1, which led to impaired mitochondrial bioenergetics and defective trans-mitochondrial cristae alignment, resulting in cardiac dysfunction [22]. In addition, SIRT3 could promote mitophagy to improve MIRI through deacetylating FOXO1 and FOXO3a [23]. Additionally, SIRT3 deficiency further worsened diabetic cardiac dysfunction by inhibiting FOXO3a-PINK1-parkin-mediated mitophagy [24]. Therefore, regulation of mitochondrial dynamics and mitophagy via SIRT3-FOXO3a signaling is of great importance to effectively improve cardiac function and ameliorate HF.

In this research, the cardioprotective effect of omentin1 released by adipose tissue on HF was investigated in vivo and in vitro, and its potential mechanisms against heart failure were further elucidated, especially focusing on mitochondrial dynamical homeostasis and mitophagy, which might provide new inspirations for the therapy of cardiovascular diseases from the crosstalk between heart and adipose tissue.

## Materials and methods

### Drugs and reagents

Recombinant human omentin1 protein was purchased from Enzo Life Sciences (New York, USA). 3-TYP, a selective SIRT3 inhibitor, was purchased from MedChemExpress (Shanghai, China). MitoTracker™ Deep Red FM was purchased from Thermo Fisher Scientific (Waltham, MA, USA; lot number: M22426). Mito-SOX red mitochondrial superoxide indicator was purchased from Warbio (Nanjing, China). Methyl thiazolyl tetrazolium (MTT) was purchased from Amresco (Washington, USA). Brain natriuretic peptide (BNP) and omentin assay kits were purchased from jin Yibai Biological Technology Co., Ltd (Nanjing, China). The assay kits of serum lactate dehydrogenase (LDH) and creatine kinase (CK) were purchased from Nanjing Jiancheng Bioengineering Institute (Nanjing, China). Cell or tissue mitochondrial isolation kit, LDH cytotoxicity assay kit, Quickblock™ blocking buffer, and mitochondrial membrane potential assay kit with JC-1 were obtained from Beyotime Biotechnology Institute (Shanghai, China). 2,3,5-Triphenyl tetrazolium chloride (TTC) was obtained from Macklin Biochemical Co., Ltd (Shanghai, China). The following primary antibodies were obtained from Proteintech Group (Wuhan, China): Dynamin-related protein 1 (Drp1, 12,957-1-AP) antibody, Optic atrophy 1 (OPA1, 27,733-1-AP) antibody, PTEN-induced putative kinase 1 (PINK1, 232,741-AP) antibody, Sirtuin 4 (SIRT4, 66,543-1-Ig) and Sirtuin 5 (SIRT5, 15,122-1-AP). Antibodies against E3 ubiquitin ligase (Parkin, SC-32282) and Sirtuin 3 (SIRT3, SC-365175) were purchased from Santa Cruz Biotechnology (Shanghai, China). Antibodies against sequestosome 1 (p62, ab109012) and voltage-dependent anion channels (VDAC, ab154856) were obtained from Abcam (Cambridge, MA, USA). Antibodies against mitofusin 2 (Mfn2, 9482S), phospho-Drp1 (Ser616) (p-Drp1, 3455S) and forkhead box O3a (FOXO3a, 2497S) were obtained from Cell Signaling Technology (Boston, MA, USA). Antibody against INTL1 (omentin1) (DF12413) was purchased from Affinity Biosciences (Nanjing, China). Antibody against β-actin (200,068-8F10) was purchased from ZenBio (Chengdu, China).

### Ethics statements

All animal experiments were conducted in accordance with the ethical policies and all experimental procedures were approved by the Animal Ethics Committee of China Pharmaceutical University and the Laboratory Animal Management Committee of Jiangsu Province (Approval No. 220193566).

Clinical serum samples were obtained from the Jiangsu Province Hospital of Traditional Chinese Medicine. Informed consent was obtained from all human research participants (No. 2019NL-089-02).

### Human samples

A total of 23 heart failure (HF) patients and 23 healthy subjects were selected from the Jiangsu Province Hospital of Traditional Chinese Medicine. A clinician comprehensively evaluated the results of electrocardiography, echocardiography and BNP (HF associated biochemical indexes) to screen out clinical HF patients. Informed consent was obtained from all human research participants and this study was guided by the Helsinki Declaration (No. 2019NL-089-02). The characteristics of heart failure patients and healthy subjects were shown in Additional file 1: Table S1.

### Animals and animal models

ICR male mice (22–24 g) were purchased from the Yangzhou University (Jiangsu, China, certificate NO. 202012586). The animals were housed in a standard space with enough food and water. The HF model induced by prolonged myocardial ischemia (MI) was constructed by ligating the left anterior descending coronary artery for 2 weeks as previously described [25]. The successful mouse model of myocardial ischemia was confirmed by ST-segment elevation on the electrocardiography. Sham-operated mice underwent the same surgical procedures without ligation. Mice were anesthetized with isoflurane. Mice were randomized into four groups (*n* = 8/group): (i) sham group (AAV-NC, 2E*10^11^ vg/mice, i.v.); (ii) model group (AAV-NC, 2E*10^11^ vg/mice, i.v.); (iii) sham + omentin1 group (AAV-omentin1, 2E*10^11^ vg/mice, i.v.); (iv) model + omentin1 group (AAV-omentin1, 2E*10^11^ vg/mice, i.v.). All AAV were intravenously injected into mice 2 weeks before CAL. And the mice were euthanized with overdose of carbon dioxide, then heart tissues were collected 2 weeks after CAL.

### Construction and infection of recombinant human AAV-omentin1

Adeno-associated virus (AAV) vectors with fat-specific promoters PTKAA (GPAAV-PTKAA-WPRE) carrying human omentin1 (AAV-omentin1) (24,538) or negative control (AAV-NC) (24537GO-AAV) were constructed by Genomeditech company (Shanghai, China). For in vivo infection, Mice were injected with AAV-omentin1 or AAV-NC (2E*10^11^ vg/mice) via the tail vein. 2 weeks later, sham surgery and coronary artery ligation (CAL) surgery were performed. The efficiency of gene transfer into adipose tissue via AAV was determined by fluorescence assay.

### TTC staining

The hearts were rapidly removed and perfused with PBS 2 weeks after CAL, and then cut into five slices perpendicular to the long axis of the heart. The slices of hearts were incubated in 1% TTC solution at 37 °C for 15 min and pictured timely. The red area of the heart stained by TTC represented viable tissue, and the staining negative areas represented infarct myocardium. The infarct areas were analyzed through Image J software (Bethesda Md, USA).

### Cardiac hypertrophy

The hearts were rapidly removed 2 weeks after CAL. Then the hearts were cleaned with PBS and pictured timely.

### Echocardiography

2 weeks after CAL, the measurement of echocardiography was performed through Vevo 3100LT system (Visual Sonics, Toronto, Canada). The following parameters were measured: left ventricle interior diameter in diastole (LVIDd), left ventricle interior diameter in systole (LVIDs), left ventricular end-diastolic volume (LVEDV), left ventricular end-systolic volume (LVESV), left ventricle volume diastole (LV Vold), left ventricle volume systole (LV Vols), left ventricle posterior wall in diastole (LVPWd), interventricular septum in diastole (IVSd). Based on these measurements, the following parameters were calculated as cardiac function indicators: left ventricular fractional shortening (LV FS) = (LVIDd—LVIDs) / LVIDd; left ventricular ejection fraction (LV EF) = (LVEDV—LVESV) / LVEDV; stroke volume (SV) = (LV Vold—LV Vols). The following parameters were calculated as cardiac hypertrophy indicators: left ventricular mass (LV Mass) = 1.053 × [(LV Vold + LVPWd + IVSd)^3^—LV Vold^3^]; relative wall thickness (RWT) = 2 × (LVPWd / LVIDd) [26].

### Enzyme-linked immunosorbent assay (ELISA)

The blood samples were collected by puncture of the retro-orbital venous plexus of mice 2 weeks after CAL and centrifuged at 3500 rpm for 11 min to isolate serum samples. The contents of CK, BNP, and omentin1 were measured using commercial kits in accordance with the manufacturer´s instructions. The ELISA assay kits of BNP and omentin1 were purchased from jin Yibai Biological Technology Co., Ltd (Nanjing, China). The assay kit of CK was purchased from Nanjing Jiancheng Bioengineering Institute (Nanjing, China).

### Histopathologic examination

The heart tissues of mice were fixed in 10% buffered paraformaldehyde solution for 25 h, embedded in paraffin, and sliced into pieces (4–5 μm thick). Then, the heart sections were stained with Masson´s trichrome or hematoxylin–eosin (H&E) and pictured timely, which were used to observe the degree of myocardial fibrosis and histopathological damage, respectively. The myocardial fibrosis was analyzed through quantification of blue-stained collagen using Image J software (Bethesda Md, USA). The criteria of pathology score of mouse heart were as follows [27]: 0 nil, intact and arranged neatly myocardial fibers, regular myocardial cells, and no inflammatory infiltration; 1–2 minimum, focal myocytes damage; 2–3 mild, small multifocal degeneration and slight inflammatory infiltration; 3–4 moderate, extensive myofibrillar degeneration, moderate cellular damage and inflammatory infiltration; 4–6 severe, massive myofibrillar degeneration and myocardial necrosis, nucleus shrinks, diffuse inflammatory cell infiltration. To avoid subjective bias causing errors, the pathologists who examined tissue slices were blind to the heart samples.

### Transmission electron microscopy

The heart tissues of mice were fixed with glutaraldehyde for 4 h. Then they were post-fixed with 0.1 mol/L cacodylate buffer with 1% osmium for 1 h, dehydrated in graded series of acetone, and lastly embedded in epoxy resin. The ultrathin sample was prepared and the ultrastructure of mice heart tissues was investigated through transmission electron microscopy (HT7800, Hitachi Ltd, Tokyo, Japan).

### Evaluation of mitophagy and damaged mitochondria

Mitochondria are made of five distinct parts: the outer mitochondrial membrane, the inner mitochondrial membrane, intermembrane space, cristae space, and mitochondrial matrix. The morphology of mitochondrial cristae directly reflects the health of the mitochondrion. The morphological appearances of healthy mitochondria are intact and sharply defined membranes and numerous and regular cristae. The morphological appearance of swollen mitochondria is warped membranes and disintegrated or swollen cristae. The morphological appearances of vacuolar mitochondria are delamination of the inner and outer mitochondrial membranes and absent cristae. Damaged mitochondria were assessed by calculating the ratio of vacuolar or swollen mitochondria to total mitochondria in the image. The initiation of mitophagy includes the formation and expansion of the phagophore, the initial sequestering compartment, which expands into a mitophagosome. Mitophagy was assessed by calculating the ratio of mitophagosome to total mitochondria in the image [28].

### Immunohistochemistry

The heart tissues of mice were acquired 2 weeks after the CAL, immersed in paraformaldehyde, embedded in paraffin, sliced into pieces (4–5 μm thick), deparaffinized, and incubated with 3% hydrogen peroxide. Then heart sections of mice were incubated for 1 h using quick-block liquid. Primary antibodies of Mfn2, OPA1, P-Drp1(Ser616), PINK1, Parkin and SIRT3 were added to the heart sections overnight at 4 °C. The heart sections of mice were maintained with the HRP-conjugated secondary antibodies at 1:200 dilution (Biogot Technology, Nanjing, China) for 1 h. After incubation with DAB and counterstained with hematoxylin, a NanoZoomer 2.0 RS (Hamamatsu, Japan) was performed to scan the heart section at 400 × magnification.

### Cell preparation and culture

Rat H9c2 cells were got from the Shanghai Institute of Cell Biology, Chinese Academy of Science (Shanghai, China) and sustained in Dulbecco’s modified Eagle medium (DMEM) containing 100 U/mL penicillin, 100 μg/mL streptomycin and 10% fetal bovine serum in a humidified atmosphere of 5% CO_2_ at 37 °C. Passage 3–10 of cells were used, and were subjected to experiments at 85–90% confluence.

### OGD injury model

To mimic the heart failure model in vitro, oxygen glucose deprivation (OGD)-injured cell model was conducted based on a previous study [29]. The OGD-injured cells were generated by maintaining cells with glucose-free DMEM and a hypoxic environment of 94% N_2_, 5% CO_2_, and 1% O_2_ for 12 h in a humidified N_2_/CO_2_ incubator at 37 °C. Meanwhile, H9c2 cells were administrated with omentin1 (300 ng/mL) or 3-TYP (50 μM) during hypoxia.

### Measurement of cell viability and LDH

H9c2 cells were seeded in a 96-well plate at a density of 5500 cells/well. Next day, after different treatments, cell viability was determined using MTT assays as reported previously [29]. The release of LDH was also measured to further evaluate the degree of cell injury. After the incubation period, the culture supernatants of H9c2 cells were used to measure the activity of LDH in compliance with the manufacturer’s instructions.

### Determination of mitochondrial membrane potential (ΔψM)

The changes in mitochondrial transmembrane potential were analyzed using 5,5′,6,6′-Tetrachloro-1,1′,3,3′-tetraethylbenzimidazolyl-carbocyanine iodide (JC-1, BD). After various treatments, the H9c2 cells were incubated with JC-1 in darkness at 37 °C for 20 min. The ΔψM dissipation was indicated by the intensity of green fluorescence emitted by JC-1 monomers. However, the intact ΔψM was represented by the red fluorescence intensity emitted by JC-1 aggregates. The fluorescence intensity was monitored through confocal laser scanning microscopy (CLSM, LSM700, Zeiss, Germany).

### Determination of mitochondrial ROS (mitoROS)

Mito-SOX red mitochondrial superoxide indicator was used to analyze the generation of mitochondrial ROS. After various treatments, the H9c2 cells were incubated with Mito-SOX red mitochondrial superoxide indicator in darkness at 37 °C for 10 min. The mitoROS was viewed using confocal laser scanning microscopy (CLSM, LSM700, Zeiss, Germany).

### Immunofluorescence staining

The heart slices of mice were fixed in paraformaldehyde, permeabilized with 0.1% Triton X-100 in PBS, blocked with 5% BSA, and incubated with a rabbit FOXO3a antibody (1:100) overnight. The H9c2 cells were maintained with MitoTracker® Deep Red (Molecular Probes) for 30 min, and then fixed, permeabilized and maintained with primary antibody against Parkin, PINK1, Mfn2, OPA1, p-Drp1(Ser616) and SIRT3 followed by incubation with corresponding fluorescent secondary antibodies and 4´,6-Diamidino-2-phenylindole (DAPI, Beyotime, Shanghai, China). Immunostaining was visualized using confocal laser scanning microscopy (CLSM, LSM700, Zeiss, Germany).

### Mitochondria isolation

Fresh minced heart tissues were cleaned with PBS, digested with trypsin to disperse tissues, and centrifuged at 600 g for 10 s to obtain precipitation. Similarly, H9c2 cells were collected by digestion with trypsin–EDTA solution and cleaned with PBS. Thereafter, the acquired tissue precipitation and collected cells were homogenized in mitochondrial separation reagent using glass homogenizer and centrifuged at 600 g for 5 min to gain supernatant, and then the supernatant was centrifuged at 11,000 g for 10 min to gain mitochondrial precipitation. Furthermore, mitochondrial precipitation was resuspended and cleaved to get mitochondrial protein using mitochondrial lysate. Finally, the concentration of mitochondrial proteins was quantified using a BCA assay kit.

### Western blot analysis

As reported [29], in 1 mM PMSF ice-cold RIPA buffer, cells were lysed and the heart tissues acquired from the margin of infarction areas were homogenized. Proteins were acquired by centrifugation at 12,000 rpm for 10 min. Thereafter, the concentration of total proteins was quantified using a BCA assay kit. Equal amount of proteins (40 μg) was separated by denaturing SDS/PAGE and transferred to PVDF membranes by electroblotting. The membranes were blocked with quick-block buffer and incubated with primary antibodies against Parkin, PINK1, p62, Mfn2, OPA1, p-Drp1(Ser616), Drp1, FOXO3a, SIRT3, SIRT4, SIRT5, β-actin and VDAC overnight, Then, the membranes were immersed in corresponding secondary antibodies for 2 h. To quantify the expression of proteins, the protein signal was detected with the ECL plus system. The bands were visualized by a ChemiDoc™ MP System (Bio-Rad), after which band density was measured by ImageJ software (Bethesda Md, USA).

### Statistical analysis

Statistical analysis was performed by GraphPad Prism 8 (La Jolla, CA, USA) software. Student’s two-tailed *t*-test and one-way analysis of variance (ANOVA) followed by Dunnett’s test were used to comparisons between two groups and among three or more groups respectively. *P* values of < 0.05 were considered significant.

## Results

### Fat-specific omentin1 overexpression ameliorated cardiac function and myocardial injury in HF mice

**Fig. 1:**
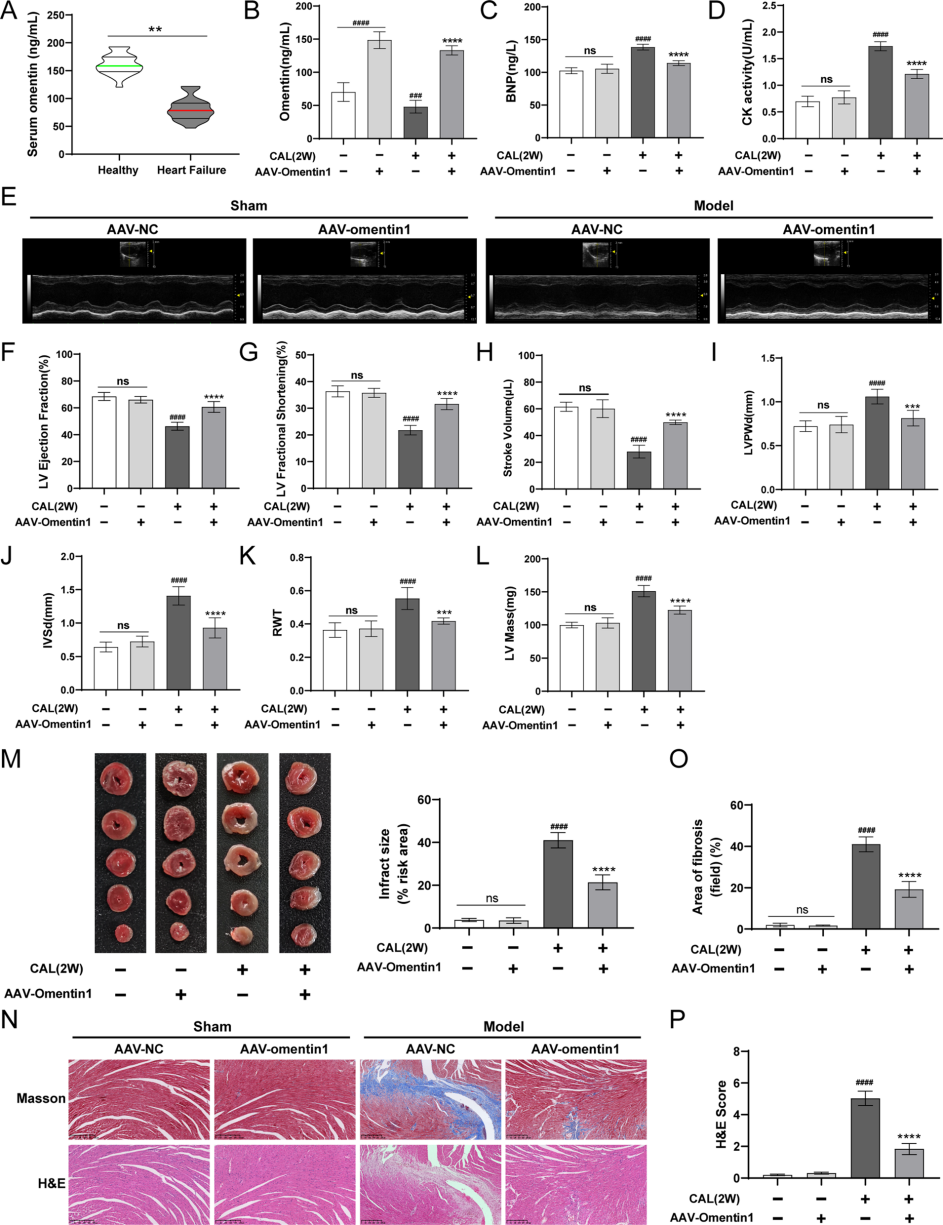
Fat-specific omentin1 overexpression ameliorated cardiac function and myocardial injury in HF mice. **A** Serum omentin1 level of heart failure (HF) patients and healthy subjects. A total of 23 HF patients and 23 healthy subjects were enrolled. **B** The serum content of omentin1 in mice with fat-specific omentin1 overexpression by intravenous injection of AAV-omentin1 and negative control by intravenous injection of AAV-NC (n = 8). **C** The serum content of BNP in mice with fat-specific omentin1 overexpression (n = 6). **D** The serum CK activity in mice with fat-specific omentin1 overexpression (n = 6). **E** Representative echocardiographs of mice with fat-specific omentin1 overexpression and the statistical results of **F** left ventricular ejection fraction (LV EF), **G** fractional shortening (LV FS), **H** stroke volume (SV), **I** left ventricle posterior wall in diastole (LVPWd), (**J**) interventricular septum in diastole (IVSd), **K** relative wall thickness (RWT) and **L** left ventricular mass (LV Mass) were presented (n = 6). **M** Representative TTC staining images of heart tissues of mice with fat-specific omentin1 overexpression and the statistical results were presented (n = 3). **N** Representative images of H&E and Masson staining of heart tissues of mice with fat-specific omentin1 overexpression and the statistical results of **O** Masson staining and **P** H&E staining were presented (n = 3), scale bar = 250 μm. Results were expressed as mean ± SD. **P < 0.01 vs the healthy subjects group; ^***####***^*P* < 0.0001 vs. the sham group; **P < 0.01, ***P < 0.001, ****P < 0.0001 vs the model group. **A** unpaired Student’s two-tailed t-test; **B–P** one-way ANOVA

As illustrated in Fig. 1A, the serum levels of omentin1 in HF patients were lower than those in healthy subjects, which further demonstrated that decreased omentin1 levels were associated with heart failure, and provided therapeutic inspiration for heart failure. The serum omentin1 content of negative control mice was about 60 ng/mL, and the mean level of serum omentin1 increased to 150 ng/mL in AAV-omentin1-treated mice at 28 days after AAV injection (Fig. 1B). In addition, the expression of omentin1 in adipose tissues in AAV-omentin1-treated mice was significantly higher than those in negative control mice, which demonstrated the successful construction of mice with fatty omentin1 overexpression (Additional file 1: Fig. S1). As shown in Fig. 1C, D, fat-specific omentin1 overexpression markedly reduced the serum BNP contents and CK activity in HF mice. Meanwhile, compared with the sham group, the cardiac function was damaged as the downregulation of LV EF, LV FS and SV in the model group. Conversely, AAV-omentin1-treated HF mice showed improvement of cardiac function (Fig. 1E, H). Additionally, in the model group, the LVPWd, IVSd, RWT, and LV Mass significantly increased (Fig. 1I–L). Correspondingly, the heart sizes of HF mice were visibly larger than those of sham-operated mice (Additional file 1: Fig. S2). All of these indicated the development of cardiac remodeling and pathological hypertrophy in HF mice. However, the fat-specific omentin1 overexpression remarkably inhibited the development of cardiac remodeling and hypertrophy. Simultaneously, HF mice appeared obviously myocardial infarct areas, and omentin1 significantly decreased infarct size in HF mice (Fig. 1M). Furthermore, the histopathological examination of heart tissues in HF mice showed observable augment of left ventricular wall fibrosis, and severely morphological damage including extensively myocardial structural destruction, irregularly arranged myocardial fibers, diffusely inflammatory infiltration, and massive myocardial necrosis. Whereas, the fat-specific omentin1 overexpression observably ameliorated cardiac pathological features (Fig. 1N–P). These results revealed the ameliorative effects of omentin1 on cardiac function and myocardial injury in HF mice.

### Fat-specific omentin1 overexpression up-regulated SIRT3-FOXO3a signaling and maintained mitochondrial dynamical homeostasis in HF mice

**Fig. 2:**
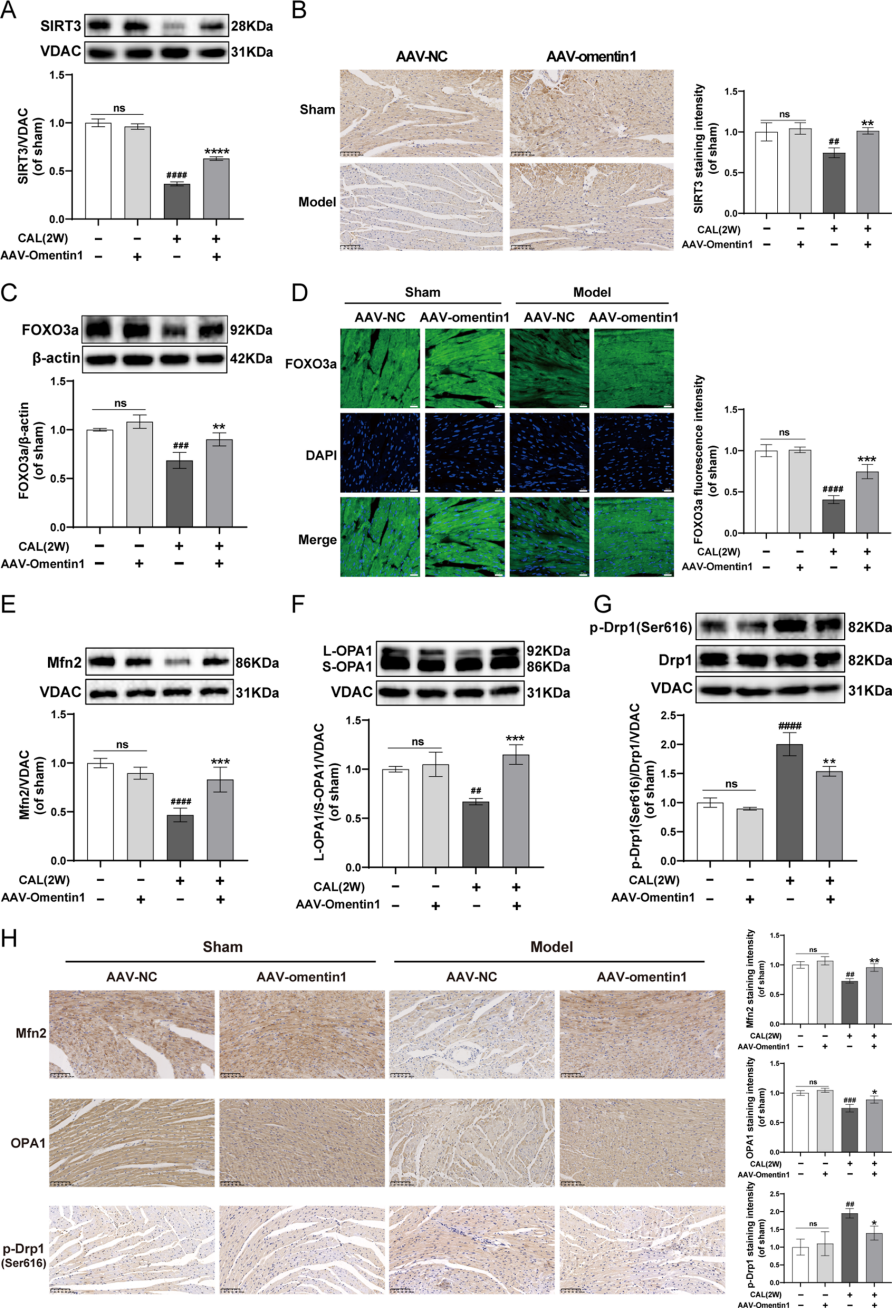
Fat-specific omentin1 overexpression up-regulated SIRT3-FOXO3a signaling and maintained mitochondrial dynamical homeostasis in HF mice. **A** Representative western blotting images of the expression of SIRT3 in myocardial mitochondria of mice with fat-specific omentin1 overexpression (n = 3). **B** Representative immunohistochemical images of the expression of SIRT3 in heart tissues of mice with fat-specific omentin1 overexpression, scale bar = 100 μm (n = 3). **C** Representative western blotting images of the expression of FOXO3a in heart tissues of mice with fat-specific omentin1 overexpression (n = 3). **D** Representative immunofluorescent images of the expression of FOXO3a in heart of mice with fat-specific omentin1 overexpression, scale bar = 20 μm (n = 3). Representative western blotting images of the expression of **E** Mfn2, **F** L-OPA1/S-OPA1 and **G** p-Drp1(Ser616)/Drp1 in myocardial mitochondria of mice with fat-specific omentin1 overexpression (n = 3). **H** Representative immunohistochemical images of the expression of Mfn2, OPA1 and p-Drp1(ser616) in heart tissues of mice with fat-specific omentin1 overexpression, scale bar = 100 μm (n = 3). Results were gained from three independent experiments and were expressed as mean ± SD. ^***##***^*P* < 0.01, ^***###***^*P* < 0.001, ^***####***^*P* < 0.0001 vs. the sham group; *P < 0.05, **P < 0.01, ***P < 0.001, ****P < 0.0001 vs. the model group. **A–H** one-way ANOVA

As exhibited in Fig. 2A–D, the MI-induced HF mice significantly decreased the expression of SIRT3 and FOXO3a compared to sham-operated mice. However, after injection of fat-specific AAV-omentin1 for 28 days, the reduced levels of SIRT3 and FOXO3a induced by HF were improved. Similarly, the immunohistochemical and immunofluorescent analysis demonstrated that fat-specific human omentin1 overexpression significantly promoted the expression of SIRT3 and FOXO3a in HF mice. Additionally, compared with sham-operated mice, the expression of SIRT4 and SIRT5 in myocardial mitochondria of HF mice were significantly decreased. While, the fat-specific omentin1 overexpression did not affect the expression of SIRT4 and SIRT5 in myocardial mitochondria of mice (Additional file 1: Fig. S3). Furthermore, the MI-induced cardiac injury led to unbalanced mitochondrial fusion-fission dynamics, as restraint of mitochondria fusion and enhancement of mitochondria fission in HF mice. While, the fat-specific omentin1 overexpression markedly increased the expression of Mfn2 and L-OPA1/S-OPA1 and decreased the level of p-Drp1(Ser616)/Drp1, which maintained mitochondrial dynamical homeostasis of myocardium in HF mice (Fig. 2E–G). Simultaneously, the results of immunohistochemistry also certified that fat-specific omentin1 overexpression dramatically mitigated unbalanced mitochondrial fusion-fission dynamics of myocardium in HF mice (Fig. 2H).

### Fat-specific omentin1 overexpression promoted mitophagy in HF mice

**Fig. 3:**
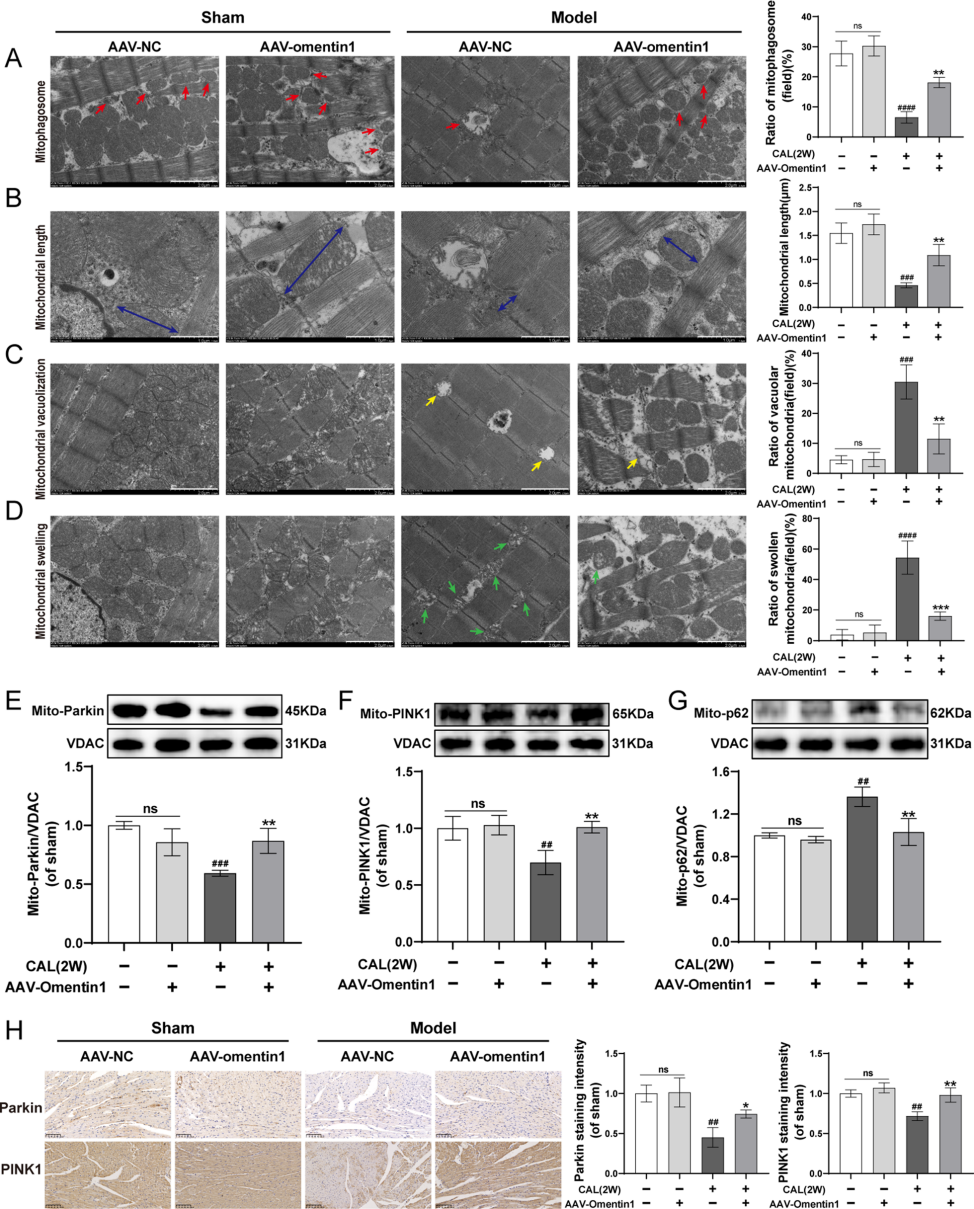
Fat-specific omentin1 overexpression promoted mitophagy in HF mice. **A** The ratio of mitophagosome (red arrows) in field total mitochondria, **B** the mitochondrial length (blue arrows), **C** the ratio of vacuolar mitochondria (yellow arrows) in field total mitochondria and **D** the ratio of swollen mitochondria (green arrows) in field total mitochondria were calculated in representative transmission electron micrographs of heart tissues of mice with fat-specific omentin1 overexpression, scale bar = 1 or 2 μm (n = 3). Representative western blotting images of the expression of **E** Parkin, **F** PINK1 and **G** p62 in myocardial mitochondria of mice with fat-specific omentin1 overexpression (n = 3). **H** Representative immunohistochemical images of the expression of Parkin and PINK1 in heart tissues of mice with fat-specific omentin1 overexpression, scale bar = 100 μm (n = 3). All experiments were performed in triplicate. Results were expressed as mean ± SD. ^***##***^*P* < 0.01, ^***###***^*P* < 0.001, ^***####***^*P* < 0.0001 vs. the sham group; *P < 0.05, **P < 0.01 vs. the model group. **A–H** one-way ANOVA

Mitophagy, a mitochondrial quality control machinery, plays a vital role in sustaining mitochondrial homeostasis. As illustrated in Fig. 3A–D, the number of mitophagosomes in HF mice was markedly lower than that of sham-operated mice. Whereas, treatment with fat-specific AAV-omentin1 remarkably enhanced the frequency of incorporation of mitochondria into autophagic vacuoles. Additionally, the mitochondrial length was shortened, and the number of vacuolar mitochondria and swollen mitochondria were increased in HF mice, which directly reflected dysfunctional and unhealthy mitochondria. However, the damaged mitochondria morphology was observably ameliorated in AAV-omentin1-treated mice. Furthermore, compared with sham-operated mice, the expression of Parkin and PINK1 were decreased, and the expression of p62 was increased in myocardial mitochondria of HF mice. While, fat-specific human omentin1 overexpression significantly induced the upregulation of mito-Parkin and mito-PINK1, and downregulation of mito-p62 in HF mice (Fig. 3E–G). Simultaneously, immunohistochemical analysis demonstrated that treatment with fat-specific AAV-omentin1 remarkably increased the expression of Parkin and PINK1 in HF mice (Fig. 3H). All of these suggested that the high level of circulating omentin1 markedly promoted cardiac mitophagy and maintained mitochondrial homeostasis in HF mice.

### Omentin1 protected OGD-injured cardiomyocytes and enhanced SIRT3-FOXO3a signaling

**Fig. 4:**
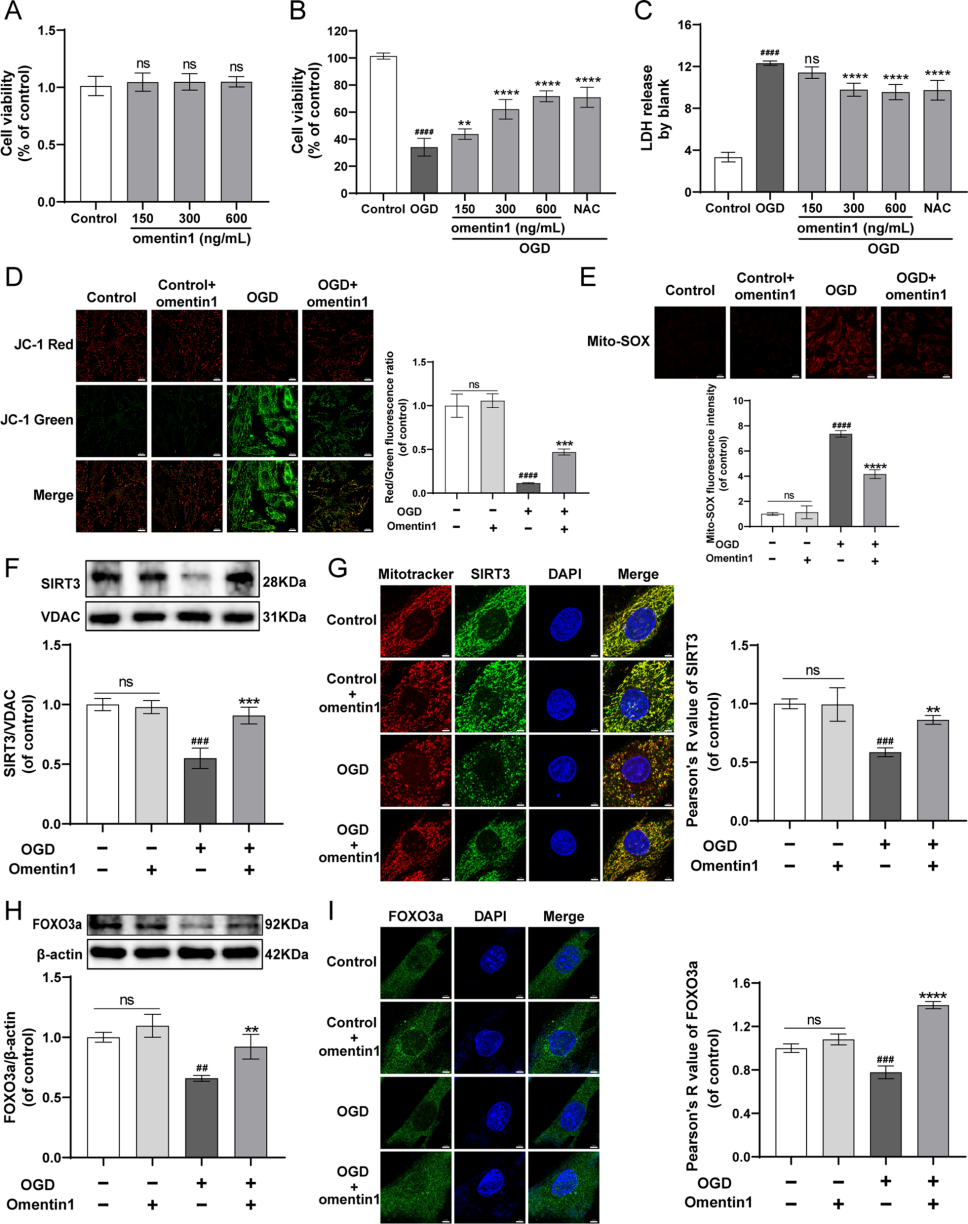
Omentin1 protected OGD-injured cardiomyocytes and enhanced SIRT3-FOXO3a signaling. **A** The cell viability of cardiomyocytes without OGD injury subjected to omentin1 were measured (n = 8). **B** The cell viability of cardiomyocytes with OGD injury subjected to omentin1 were measured (n = 8). **C** The LDH release of cardiomyocytes with OGD injury subjected to omentin1 were measured (n = 8). **D** Representative images of mitochondrial membrane potential (ΔψM) were presented, scale bar = 20 μm (n = 3). Green fluorescence represents the dissipation of ΔψM. Red fluorescence represents intact ΔψM. **E** Representative images of mitochondrial SOX were presented, which represents the generation of mitochondria-derived ROS, scale bar = 20 μm (n = 3). **F** Representative western blotting images of the expression of SIRT3 in cardiomyocytes treated with omentin1 (n = 3). **G** Representative immunofluorescent images of the expression of SIRT3 in mitochondria of cardiomyocytes treated with omentin1 and the statistical results using quantitative analysis of Pearson’s correlation coefficient (PCC) were presented, scale bar = 5 μm (n = 3). **H** Representative western blotting images of the expression of FOXO3a in cardiomyocytes treated with omentin1 (n = 3). **I** Representative immunofluorescent images of the expression of FOXO3a in nucleus of cardiomyocytes treated with omentin1 and the statistical results using quantitative analysis of PCC were presented, scale bar = 5 μm (n = 3). Results were gained from three independent experiments and were expressed as mean ± SD. ^***##***^*P* < 0.01, ^***###***^*P* < 0.001, ^***####***^*P* < 0.001 vs. the control group; **P < 0.01, ***P < 0.001, ****P < 0.0001 vs. the OGD group. **A–I** one-way ANOVA

As shown in Fig. 4A–C, in contrast to negative control cardiomyocytes, 150–600 ng/mL of omentin1 had no effect on the cell viability of cardiomyocytes without OGD injury. Whereas the viability of OGD-injured cardiomyocytes subjected to 150–600 ng/mL omentin1 was observably enhanced, and omentin1 treatment restrained the release of LDH. Therefore, 300 ng/mL omentin1 was used for further experiments. Moreover, 300 ng/mL omentin1 treatment significantly diminished the dissipation of mitochondrial membrane potential (ΔψM) and the generation of mitoROS induced by OGD injury (Fig. 4D–E). Additionally, the results of western blotting and immunofluorescent analysis indicated that OGD injury markedly reduced the expression of SIRT3 and the colocalization of SIRT3 with mitochondria. However, omentin1 treatment markedly induced upregulation of SIRT3 and enhanced the localization of SIRT3 at mitochondria in OGD-injured cardiomyocytes. And omentin1 did not affect the cardiomyocytes without OGD damage (Fig. 4F–G). Furthermore, in OGD-injured cardiomyocytes, omentin1 treatment significantly increased the expression of FOXO3a and the nucleus accumulation of FOXO3a using Pearson’s correlation coefficient (PCC) which is a statistic for quantifying colocalization [30] (Fig. 4H–I).

### Omentin1 attenuated OGD-induced unbalanced mitochondrial fusion-fission dynamics of cardiomyocytes

**Fig. 5:**
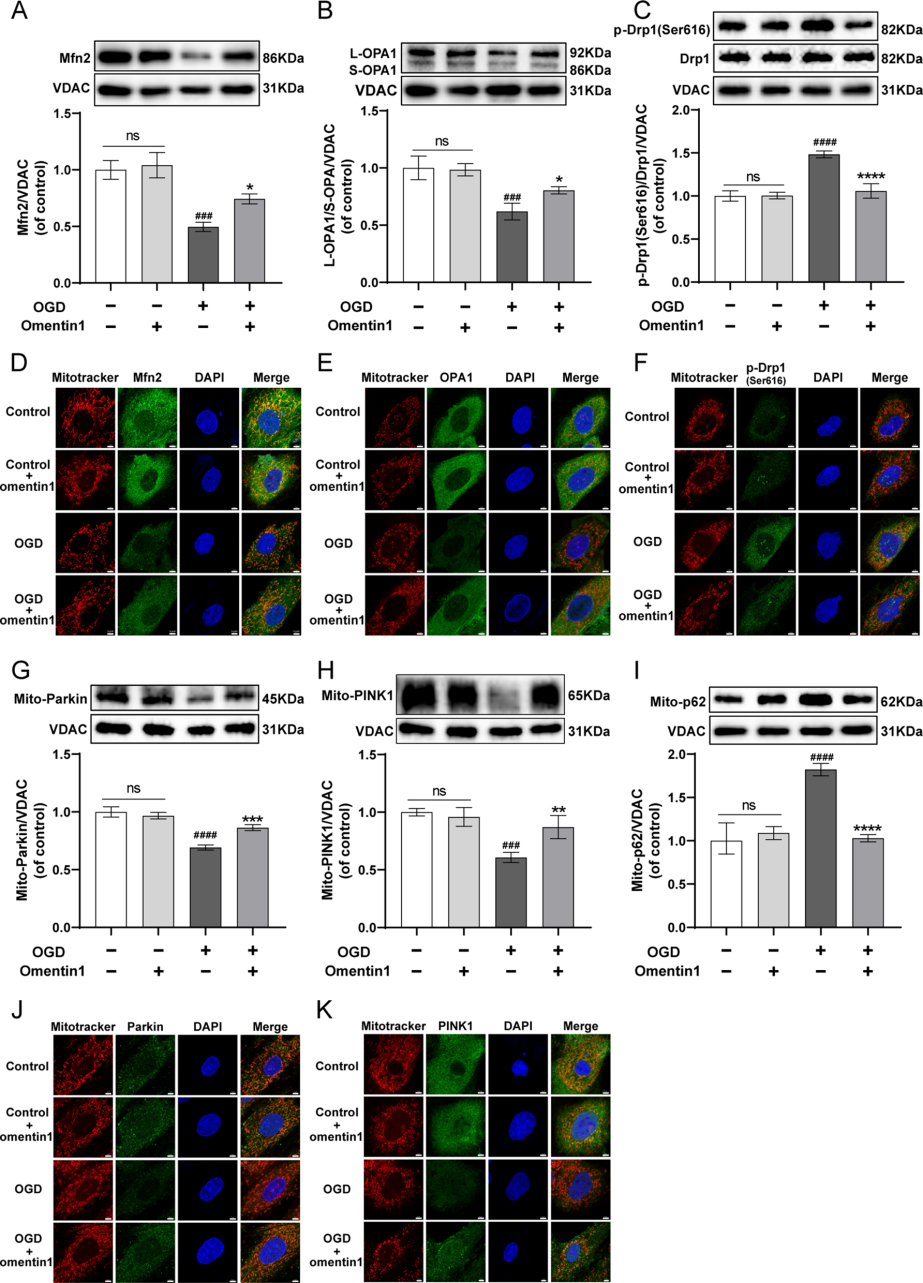
omentin1 attenuated OGD-induced unbalanced mitochondrial fusion-fission dynamics and enhanced mitophagy in OGD-injured cardiomyocytes. Representative western blotting images of the expression of **A** Mfn2, **B** L-OPA1/S-OPA1 and **C** p-Drp1(Ser616)/Drp1 in mitochondria of cardiomyocytes treated with omentin1 (n = 3). Representative immunofluorescent images of the expression of **D** Mfn2, **E** OPA1 and **F** p-Drp1(Ser616) in mitochondria of cardiomyocytes treated with omentin1, scale bar = 5 μm (n = 3). Representative western blotting images of the expression of **G** Parkin, **H** PINK1 and **I** p62 in mitochondria of cardiomyocytes treated with omentin1 (n = 3). Representative immunofluorescent images of the expression of **J** Parkin and **K** PINK1 in mitochondria of cardiomyocytes treated with omentin1, scale bar = 5 μm (n = 3). Results were gained from three independent experiments and were expressed as mean ± SD. ^**###**^*P* < 0.001, ^**####**^*P* < 0.0001 vs. the control group; *P < 0.05, **P < 0.01, ***P < 0.001, ****P < 0.0001 vs. the OGD group. **A–I**: one-way ANOVA

The results of western blotting analysis showed that OGD injury resulted in unbalanced mitochondrial fusion-fission dynamics, as the significantly down-regulated expression of Mfn2 and L-OPA1/S-OPA1 and upregulation of p-Drp1(Ser616)/Drp1 in mitochondria of cardiomyocytes. However, omentin1 treatment markedly enhanced mitochondria fusion, inhibited mitochondria fission and sustained mitochondrial dynamical homeostasis in OGD-injured cardiomyocytes (Fig. 5A–C). Simultaneously, immunofluorescence analysis indicated that omentin1 treatment observably increased the localization of Mfn2 and OPA1 in mitochondria and decreased mitochondria accumulation of p-Drp1(Ser616) in OGD-injured cardiomyocytes using quantitative analysis of PCC. Additionally, omentin1 treatment observably attenuated damaged mitochondria morphology that mitochondrial structures of short tubular or fragmentation turned into elongated tubular or interconnected network (Fig. 5D–F, Additional file 1: Fig. S4A–C). In conclusion, omentin1 effectively maintained mitochondrial dynamical homeostasis and ameliorated the damaged mitochondria morphology in OGD-injured cardiomyocytes.

### Omentin1 enhanced mitophagy in OGD-injured cardiomyocytes

As illustrated in Fig. 5G–I, compared with the control group, the expression of mito-Parkin, mito-PINK1 were significantly decreased and the mito-p62 expression was increased in the OGD group. However, 12 h after the omentin1 treatment, the translocation of Parkin from cytosol to mitochondria was markedly enhanced, the level of PINK1 was increased and p62 was diminished in mitochondrial. And omentin1 did not affect H9c2 cardiomyocytes without OGD injury. Similarly, the OGD injury remarkably inhibited the colocalization of the mitochondria with Parkin and PINK1. But omentin1 treatment observably augmented the localization of Parkin and PINK1 in mitochondria. Moreover, less mitochondrial fragmentation indicated that omentin1 partially ameliorated the damaged mitochondria morphology induced by OGD injury (Fig. 5J–K, Additional file 1: Fig. S4D–E). These results demonstrated that mitophagy was inhibited in cardiomyocytes with 12 h-OGD injury, and omentin1 promoted mitophagy in OGD-injured cardiomyocytes.

### SIRT3 participated in the regulation effect of omentin1 on unbalanced mitochondrial fusion-fission dynamics and mitophagy

**Fig. 6:**
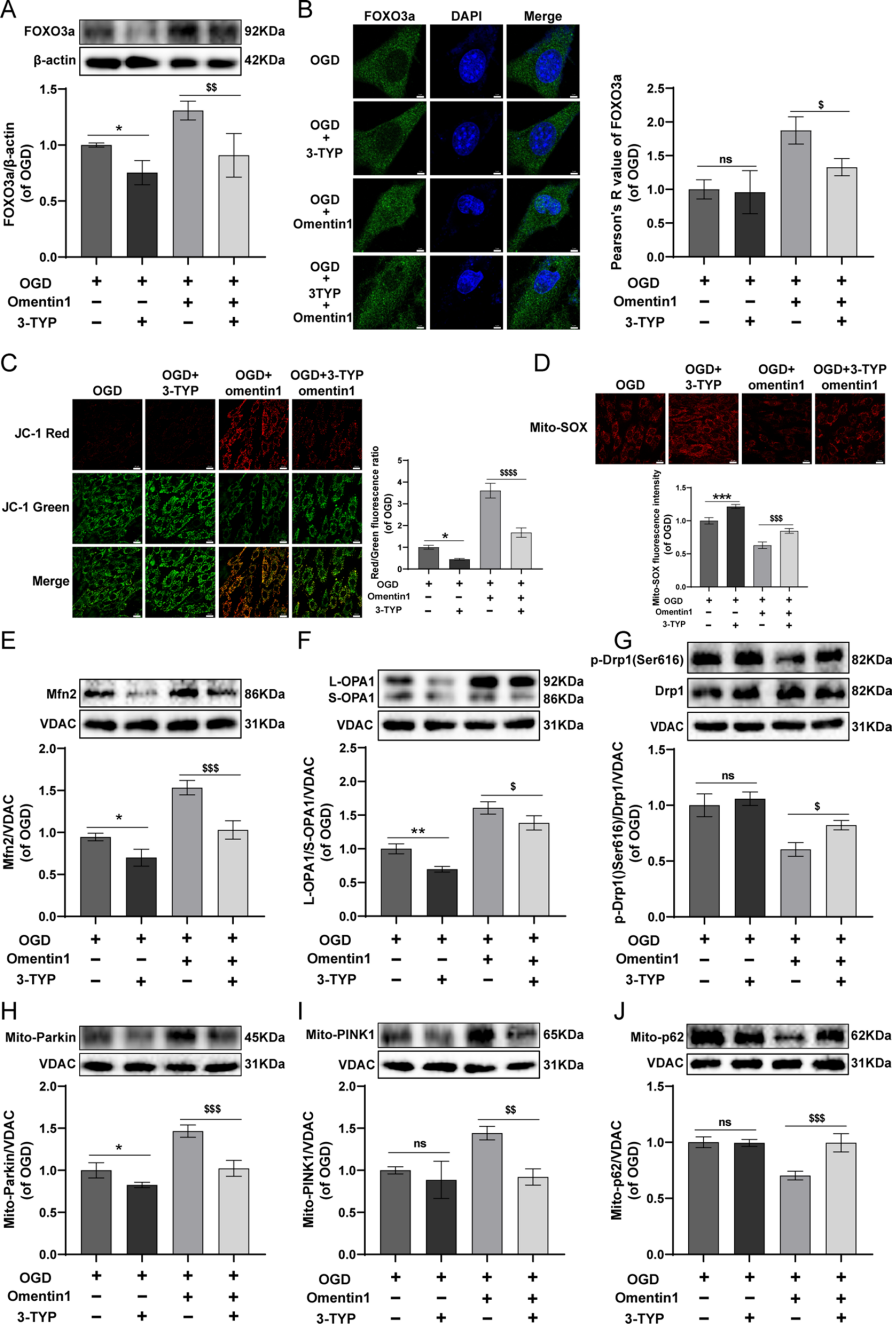
SIRT3 participated in the regulation effect of omentin1 on unbalanced mitochondrial fusion-fission dynamics and mitophagy. **A** Representative images of western blotting analysis of the expression of FOXO3a in cardiomyocytes treated with omentin1 and 3-TYP (n = 3). **B** Representative images of immunofluorescent analysis of the expression of FOXO3a in nucleus of cardiomyocytes treated with omentin1 and 3-TYP and the statistical results using quantitative analysis of Pearson’s correlation coefficient were presented, scale bar = 5 μm (n = 3). **C** Representative images of mitochondrial membrane potential (ΔψM) were presented, scale bar = 20 μm. Green fluorescence represents the dissipation of ΔψM. Red fluorescence represents intact ΔψM (n = 3). **D** Representative images of mitochondrial SOX were presented, which represents the generation of mitochondria-derived ROS, scale bar = 20 μm (n = 3). Representative images of western blotting analysis of the expression of **E** Mfn2, **F** L-OPA1/S-OPA1 and **G** p-Drp1(Ser616)/Drp1 in mitochondria of cardiomyocytes treated with omentin1 and 3-TYP (n = 3). Representative images of western blotting analysis of the expression of **H** Parkin, **I** PINK1 and **J** p62 in mitochondria of cardiomyocytes treated with omentin1 and 3-TYP (n = 3). Results were gained from three independent experiments and were expressed as mean ± SD. *P < 0.05, ***P < 0.001 vs. the OGD group; ^*$*^*P* < 0.05, ^*$$*^*P* < 0.01, ^*$$$*^*P* < 0.001, ^*$$$$*^*P* < 0.0001 vs. the OGD + omentin1 group. **A–J** one-way ANOVA

**Fig. 7:**
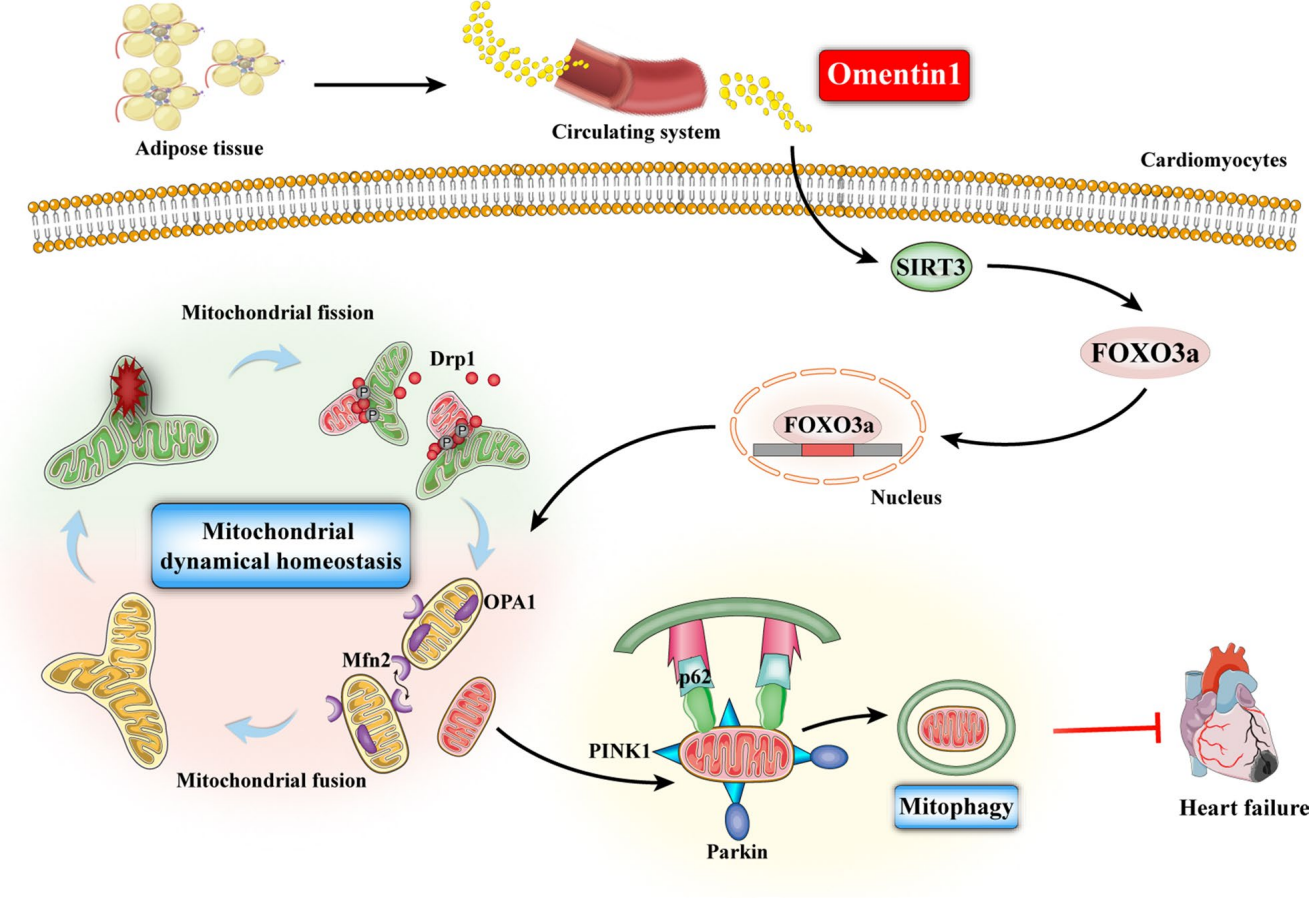
The schematic of omentin1 ameliorated ischemia-induced HF via maintaining mitochondrial dynamical homeostasis and mitophagy

To confirm whether SIRT3 is involved in the regulation effect of omentin1 on mitochondrial homeostasis, 3-TYP, a specific inhibitor of SIRT3, was performed to further verify. The expression of SIRT3 in 3-TYP-treated cardiomyocytes was significantly lower than that in 3-TYP-untreated cardiomyocytes, which demonstrated the high inhibitory effect of 3-TYP on SIRT3 activity (Additional file 1: Fig. S5). As shown in Fig. 6A–B, omentin1-induced upregulation of FOXO3a expression and nucleus transduction of FOXO3a were dramatically abolished upon the treatment with 3-TYP in OGD-injured cardiomyocytes. Additionally, only 3-TYP treatment decreased the expression of FOXO3a in OGD-injured cardiomyocytes. Moreover, compared with OGD + omentin1 group, 3-TYP treatment observably enhanced the dissipation of ΔψM and the generation of mitoROS in OGD-injured cardiomyocytes treated with omentin1 (Fig. 6C, D). Besides, 3-TYP treatment markedly diminished the level of Mfn2 and L-OPA1/S-OPA1 and enhanced p-Drp1(Ser616)/Drp1 expression in OGD-injured cardiomyocytes treated with omentin1, thus leading to unbalanced mitochondrial fusion-fission dynamics (Fig. 6E–G). Next, the mitophagy activated by omentin1 was significantly inhibited by 3-TYP in OGD-injured cardiomyocytes, as the down-regulated expression of mito-Parkin and mito-PINK1 and the upregulation of mito-p62 in OGD-injured cardiomyocytes treated with omentin1 and 3-TYP (Fig. 6H–J). These results suggested that SIRT3 exerted an essential role in the restorative effect of omentin1 for mitochondrial homeostasis. Taken together, the schematic of omentin1 ameliorated ischemia-induced HF via maintaining mitochondrial homeostasis was presented in Fig. 7.

## Discussion

Heart failure is characterized by high morbidity and mortality worldwide, which is caused by the long-term insufficient blood supply to heart induced by coronary heart disease, myocardial infarction, obesity and the like, and it is the end-stage of various diseases. With the development of system biology, it would be more effective to treat complex multi-factorial diseases from the perspective of tissue crosstalk. Adipose tissue, as crucial energy storage and endocrine organ, not only provides energy but also secretes multiple adipocytokines, such as adiponectin, resistin and the like, to regulate various metabolic tissues. Additionally, the dysfunction of adipose tissue in the synthesis or release of adipocytokines could induce the underlying pathogenesis of both obesity and HF [31]. Simultaneously, hypoxic adipose tissue altered adipocytokine secretion, caused skeletal muscle atrophy, cardiovascular remodeling and endothelial dysfunction, and developed ultimately HF [32].

Omentin, an adipokine expressed by adipose tissue and released into the bloodstream, was found to provide certain therapeutic effects on complex diseases such as osteoporosis, acute lung injury, arterial calcification, and MI/R injury [14]. The majority of experimental studies have pointed to favorable effects of omentin on cardiometabolic risk. Preclinical studies demonstrated that omentin prevented the activation and senescence of vascular endothelial cells [33], diminished the calcification and proliferation of vascular smooth muscle cells [34], curbed macrophage infiltration and foam cell formation [35], and maintained cardiomyocyte function to promote cardiovascular health by improving cardiac vasorelaxation and preventing atherosclerosis [36, 37]. Consistent with the preclinical studies, circulating omentin1 concentration was increased by improving insulin sensitivity after weight loss, and circulating omentin1 levels were inversely connected with atherosclerosis, carotid plaque, and left ventricular diastolic function [38, 39]. However, in contrast to the above studies, prospective studies demonstrated that higher omentin1 concentrations were connected with increased cardiovascular risk in diabetes patients and angiographied coronary patients [15, 40]. All of these showed that molecular interactions between omentin1 and cardiovascular risk factors are complex. Elevated omentin levels in individuals at high risk of cardiovascular events could reflect a counter regulatory mechanism. Omentin might be upregulated in response to metabolic and inflammatory stimuli that contributed to atherogenesis [15]. Therefore, the biological effects of omentin1 on different cardiovascular diseases under different metabolic conditions need to be further studied. Whereas, the improvement of omentin1 on cardiac function and HF is not yet clear, and its potential mechanism still needs to be further elucidated. In the present study, clinically, the serum omentin1 level was down-regulated in HF patients compared with healthy subjects. Increased circulating omentin1 level via fat-specific overexpression observably ameliorated cardiac function, cardiac hypertrophy, infarct size and cardiac pathological features in HF mice. And treatment with recombinant human omentin1 protein markedly enhanced OGD-injured cardiomyocytes viability and mitochondrial membrane potential (ΔψM), and restrained LDH release and the generation of mitoROS.

It is known that prolonged MI can provoke mitochondrial damage, and mitochondrial homeostasis is disrupted during the development of HF, which leads to inadequate supplement of ATP and destruction of the cellular energy network [41]. Therefore, controlling mitochondrial quality through periodically mitochondrial fusion, fission and mitophagy is the key to maintaining mitochondrial homeostasis [42]. Mitochondrial dynamics is important to compensate mitochondrial damage and to eliminate irreparably damaged mitochondria through mitochondrial fusion and fission, respectively, and its disorders are implicated in neoplastic, endocrine, neurodegenerative and cardiovascular diseases [43]. Mitochondrial fission is regulated by Drp1, cytoplasmic guanosine triphosphatases (GTPases) which translocates to the outer mitochondrial membrane (OMM) upon phosphorylation of serine 616 [44]. Studies have shown that I/R injury led to cardiomyocyte death through the enhancement of mitochondrial fission mediated by p-Drp1(Ser616) and oxidative stress injury [45]. Moreover, pharmacological inhibition of the Drp1 activation reduced heart infarct areas and protected cardiomyocytes against MI injury [46]. Mitochondrial fusion is accomplished by cooperative work of Mfn1 and Mfn2 which are located in OMM and orchestrate OMM fusion. While optic atrophy1 (OPA1) is located in the inner mitochondrial membrane (IMM), which is required for IMM fusion [47]. Previous studies have proved that mitochondrial fusion is essential for the maintenance of normal mitochondrial morphology, respiratory and contractile functions of the adult heart [48]. Similarly, deficiency of Mfn2 leads to depletion of mitochondrial coenzyme Q, which impairs the capacity of the mitochondrial respiratory chain [49]. Additionally, since mitochondrial fusion is only dependent on the longer OPA1 form (L-OPA1) and mitochondrial fission is related to the shorter OPA1 form (S-OPA1), the balanced accumulation of both OPA1 forms serves as the center for coordinating mitochondrial fusion, fission, and sustaining normal mitochondrial morphology [50, 51]. Several studies have reported that the level of OPA1 was diminished in heart samples of both rats with HF, accompanied by smaller and fragmented mitochondria [52]. However, the mild overexpression of OPA1 attenuated heart I/R injury, denervation-induced muscular atrophy, and the susceptibility of Fas-induced liver injury through the resistance of mitochondrial apoptotic cristae remodeling [53]. In our present study, fat-specific human omentin1 overexpression and administration of recombinant human omentin1 protein increased mitochondrial fusion and decreased mitochondrial fission, thus remarkably improving damaged mitochondria morphology and unbalanced mitochondrial fusion-fission dynamics in HF mice and OGD-injured cardiomyocytes.

Mitophagy, selective autophagy specific to the degradation of mitochondria, is vital for mitochondrial quality control [54]. The best-characterized pathway of mitophagy is PINK1/Parkin signaling. A study has shown that the PINK1/Parkin-mediated mitophagy was inhibited in advanced dystrophic cardiomyopathy [55]. Being subsistent in the cytosol at baseline conditions, Parkin rapidly translocates into OMM and ubiquitinates several targets on damaged mitochondria when mitochondria lose membrane potential. Then p62, a ubiquitin-binding protein, binds ubiquitinated proteins to LC3, which promotes the isolation of damaged mitochondria into the autophagosome [56]. Multiple studies have demonstrated that mice deficient in PINK1 are more susceptible to HF under cardiac stress overload and heart I/R injury [57]. In our present study, fat-specific omentin1 overexpression augmented the expression of mito-Parkin, mito-PINK1 and restraint of mito-p62 expression, accompanied by the increased number of mitophagosomes in HF mice*.* Simultaneously, omentin1 enhanced the expression and localization of Parkin and PINK1 at mitochondria in OGD-injured cardiomyocytes. Taken together, omentin1 promoted PINK1/Parkin-dependent mitophagy and controlled mitochondrial quality.

SIRT3, a mitochondrial energy sensor, is regulated by metabolic co-factor NAD^+^ and nicotinamide [58]. Meanwhile, SIRT3 sustains mitochondrial homeostasis through regulating acetylation modification of its substrates which are involved in the comprehensive field of mitochondrial biological function [59]. Transcribed in the nucleus, inactive SIRT3 is proteolytically cleaved to the active form when it is translocated into mitochondria [60]. As a guardian of mitochondrial homeostasis, the deficiency of SIRT3 destroyed the systolic function of heart [61]. Besides, in heart failure, the downregulation of SIRT3 disturbed oxidative phosphorylation and resulted in the energy metabolism imbalance of cardiomyocytes [62]. Furthermore, the SIRT3 substrate FOXO3a regulated apoptosis and mitophagy, and SIRT3 activated its activity and nuclear translocation, thereby enhancing antioxidant defense against cardiac hypertrophy [63]. Another major regulator of mitochondrial homeostasis is mammalian target of rapamycin (mTOR) which is involved in many cellular processes, such as cell growth, metabolism, proliferation, and apoptosis [64]. mTOR controlled mitochondrial biogenesis and respiration via stimulating expression of mitochondrial proteins and inhibited mitochondrial degradation by suppressing autophagy [65]. Meanwhile, mTOR stimulated mitochondrial fission through MTFP1, which induced mitochondrial fragmentation, regulated mitochondrial morphology, and controlled cell survival [66]. In addition, mTOR and AMPK played an important role in balancing cellular energy homeostasis by sensing cellular ATP levels and were key upstream regulators for triggering autophagy [67]. Therefore, mTOR act as a central regulator of mitochondrial dysfunction. Interestingly, several studies have shown that SIRT3 promoted autophagy by regulating AMPK-mTOR signaling, thereby ameliorating acute kidney injury, ischemic neuronal injury, diabetes and osteoarthritis [68–71]. All of these suggested that SIRT3 might be a key upstream factor regulating mitochondria, and exerted important roles in regulating mitochondrial homeostasis. In our present study, human omentin1 enhanced the expression of SIRT3, FOXO3a, and the colocalization of SIRT3 and FOXO3a with mitochondria and nucleus, respectively, in HF mice and OGD-injured cardiomyocytes. Nevertheless, 3-TYP, a selective SIRT3 inhibitor, dampened omentin1-induced upregulation of FOXO3a and nucleus transduction of FOXO3a, and inhibited the protection of omentin1 on mitochondrial function in OGD-injured cardiomyocytes. Additionally, it also degraded the ameliorative effect of omentin1 on the unbalanced mitochondrial fusion-fission dynamics, as evidenced by the inhibition of mitochondrial fusion and the augment of mitochondrial fission. And the omentin1-induced mitophagy activation was also inhibited by 3-TYP. All of these suggested that SIRT3 participated in the protective effect of omentin1 on mitochondrial homeostasis. Moreover, SIRT3, an important regulator of mitochondrial antioxidant defense mechanism, scavenged mitoROS by stimulating deacetylation of superoxide dismutase 2, thus improving mitochondrial oxidative stress damage including essential hypertension, cardiac fibrosis, and diabetic cardiomyopathy [72–74]. Meanwhile, activation of the SIRT3-FOXO3a pathway could protect mitochondria from oxidative stress, thereby ameliorating lung senescence and neurodegenerative diseases [75, 76]. Additionally, current knowledge has proved that antioxidant effects were closely related to sufficient mitochondrial biogenesis, that was, the mitoROS production could be reduced by elevating mitochondrial efficiency [77]. Therefore, omentin1 may improve mitochondrial oxidative stress damage-related diseases through SIRT3-mediated antioxidant pathways and enhanced mitochondrial biogenesis, which will provide novel ideas for the development of antioxidant drugs and elucidation of the association of complex diseases.

However, although the above results have revealed that omentin1 might ameliorate MI-induced HF through sustaining mitochondrial homeostasis via modulating SIRT3/FOXO3a signaling, further confirmation is required using SIRT3-deficient mice. Additionally, we need to further explore how omentin1 promotes the expression of SIRT3 and elucidate its possible acting site on mitochondria.

## Conclusions

Omentin1 maintained mitochondrial dynamical homeostasis and promoted PINK1/Parkin-dependent mitophagy via SIRT3/FOXO3a signaling, thereby improving MI-induced HF and enhancing the resistance of myocardium to prolonged ischemia injury. This study might lay the foundation for further application of omentin1 in cardiovascular diseases, and provide new strategies for the prevention and therapy of complex cardiovascular complications of obesity, diabetes and hypertension from the crosstalk between the heart and adipose tissue.

## Supplementary Information

**Additional file1:** **Figure S1.** Representative immunohistochemical images of the expression of omentin1 in adipose tissues of mice injected with AAV-NC or AAV-omentin1, and the statistical results of staining intensity of omentin1 were presented, scale bar = 250 μm (n = 3). The experiments were performed in triplicate. Results were expressed as mean ± SD. ^*##*^*P* < 0.01, ^*####*^*P* < 0.0001 vs. the sham group; ****P < 0.0001 vs. the model group, one-way ANOVA. **Figure S2.** Representative images of hearts of mice injected with AAV-omentin1 or AAV-NC were presented, scale bar = 1 cm (n = 6). **Figure S3.** Representative western blotting images of the expression of **A** SIRT4 and **B** SIRT5 in myocardial mitochondria of mice with fat-specific omentin1 overexpression, and the statistical results were presented (n = 4). Results were gained from three independent experiments and were expressed as mean ± SD. ^***##***^*P *< 0.01, ^***####***^*P *< 0.0001 vs. the sham group. **A-B** one-way ANOVA. **Figure S4.** The statistical results of immunofluorescence analysis of Figure 5. Pearson’s correlation coefficient represents the degree of colocalization of **A** Mfn2, **B** OPA1, **C** p-Drp1(Ser616), **D** Parkin and **E** PINK1 with mitochondria (n = 3). Results were gained from three independent experiments and were expressed as mean ± SD. ^*##*^*P* < 0.01, ^*####*^*P* < 0.0001 vs. the control group; *P < 0.05, **P < 0.01, ***P < 0.001 vs. the OGD group. **A-E** one-way ANOVA. **Figure S5.** Representative images of western blotting analysis of the SIRT3 expression in mitochondria of cardiomyocytes treated with omentin1 and 3-TYP and the statistical result was presented (n = 3). Results were gained from three independent experiments and were expressed as mean ± SD. *P < 0.05 vs. the OGD group; ^*$$$*^*P <* 0.001 vs. the OGD + omentin1 group. One-way ANOVA. **Table S1.** The clinical characteristics of heart failure (HF) patients and healthy subjects

## Data Availability

All data generated or analyzed during this study are included in this published article.

## Abbreviations

AAV: Adeno-associated virus
BNP: Brain natriuretic peptide
CAL: Coronary artery ligation
CK: Creatine kinase
Drp1: Dynamin-related protein 1
FOXO3a: Forkhead box O3a
HF: Heart failure
H&E: Hematoxylin-eosin
LDH: Lactate dehydrogenase
Mfn2: Mitofusin 2
MI: Myocardial ischemia
MIRI: Myocardial ischemia reperfusion injury
mitoROS: Mitochondrial ROS
mTOR: Mammalian target of rapamycin
MTT: Methyl thiazolyl tetrazolium
OGD: Oxygen glucose deprivation
OPA1: Optic atrophy 1
p-Drp1 (Ser616): phospho-Drp1 (Ser616)
PINK1: PTEN-induced putative kinase 1
Parkin: E3 ubiquitin ligase
p62: Sequestosome 1
SIRT3: Sirtuin 3
SIRT4: Sirtuin 4
SIRT5: Sirtuin 5
VDAC: Voltage-dependent anion channels
ΔψM: Mitochondrial membrane potential
