# Supplementary material for: Omentin1 ameliorates myocardial ischemia-induced heart failure via SIRT3/FOXO3a-dependent mitochondrial dynamical homeostasis and mitophagy
Source: J Transl Med. 2022 Oct 4;20:447. doi: 10.1186/s12967-022-03642-x (PMC9531426; doi:10.1186/s12967-022-03642-x)
Supplement: Supplementary file 1 — Additional file1: Figure S1. Representative immunohistochemical images of the expression of omentin1 in adipose tissues of mice injected with AAV-NC or AAV-omentin1, and the statistical results of staining intensity of omentin1 were presented, scale bar = 250 μm (n = 3). The experiments were performed in triplicate. Results were expressed as mean ± SD. ##P < 0.01, ####P < 0.0001 vs. the sham group; ****P < 0.0001 vs. the model group, one-way ANOVA. Figure S2. Representative images of hearts of mice injected with AAV-omentin1 or AAV-NC were presented, scale bar = 1 cm (n = 6). Figure S3. Representative western blotting images of the expression of A SIRT4 and B SIRT5 in myocardial mitochondria of mice with fat-specific omentin1 overexpression, and the statistical results were presented (n = 4). Results were gained from three independent experiments and were expressed as mean ± SD. ##P < 0.01, ####P < 0.0001 vs. the sham group. A-B one-way ANOVA. Figure S4. The statistical results of immunofluorescence analysis of Figure 5. Pearson’s correlation coefficient represents the degree of colocalization of A Mfn2, B OPA1, C p-Drp1(Ser616), D Parkin and E PINK1 with mitochondria (n = 3). Results were gained from three independent experiments and were expressed as mean ± SD. ##P < 0.01, ####P < 0.0001 vs. the control group; *P < 0.05, **P < 0.01, ***P < 0.001 vs. the OGD group. A-E one-way ANOVA. Figure S5. Representative images of western blotting analysis of the SIRT3 expression in mitochondria of cardiomyocytes treated with omentin1 and 3-TYP and the statistical result was presented (n = 3). Results were gained from three independent experiments and were expressed as mean ± SD. *P < 0.05 vs. the OGD group; $$$P < 0.001 vs. the OGD + omentin1 group. One-way ANOVA. Table S1. The clinical characteristics of heart failure (HF) patients and healthy subjects [file 12967_2022_3642_MOESM1_ESM.doc]

**Supplementary Figures**


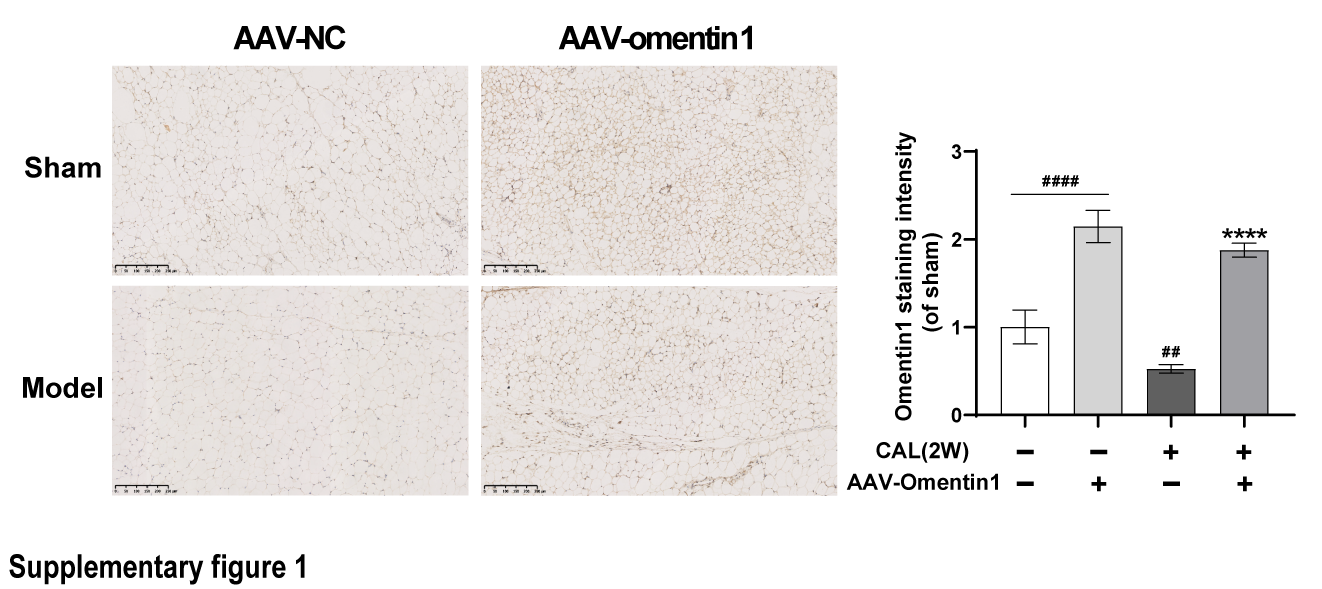


**Fig. S1** Representative immunohistochemical images of the expression of omentin1 in adipose tissues of mice injected with AAV-NC or AAV-omentin1, and the statistical results of staining intensity of omentin1 were presented, scale bar = 250 μm (n = 3). The experiments were performed in triplicate. Results were expressed as mean ± SD. ***^##^****P* < 0.01, ***^####^****P* < 0.0001 vs. the sham group; *****P* < 0.0001 vs. the model group, one-way ANOVA.


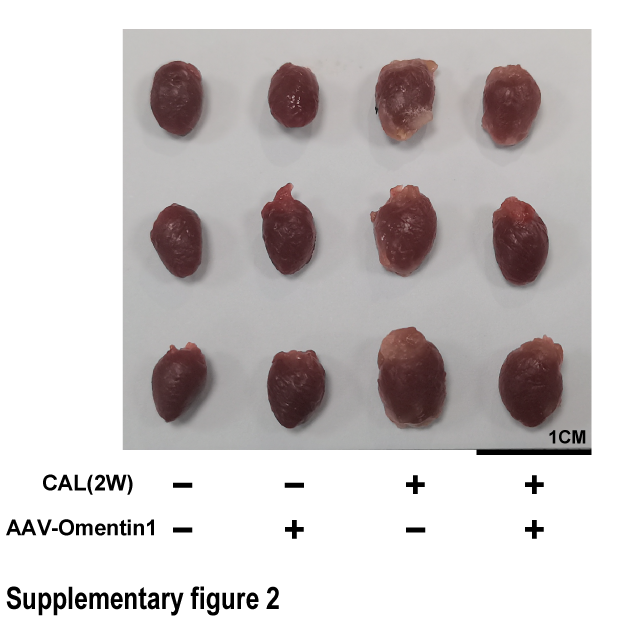


**Fig. S2** Representative images of hearts of mice injected with AAV-omentin1 or AAV-NC were presented, scale bar = 1 cm (n = 6).


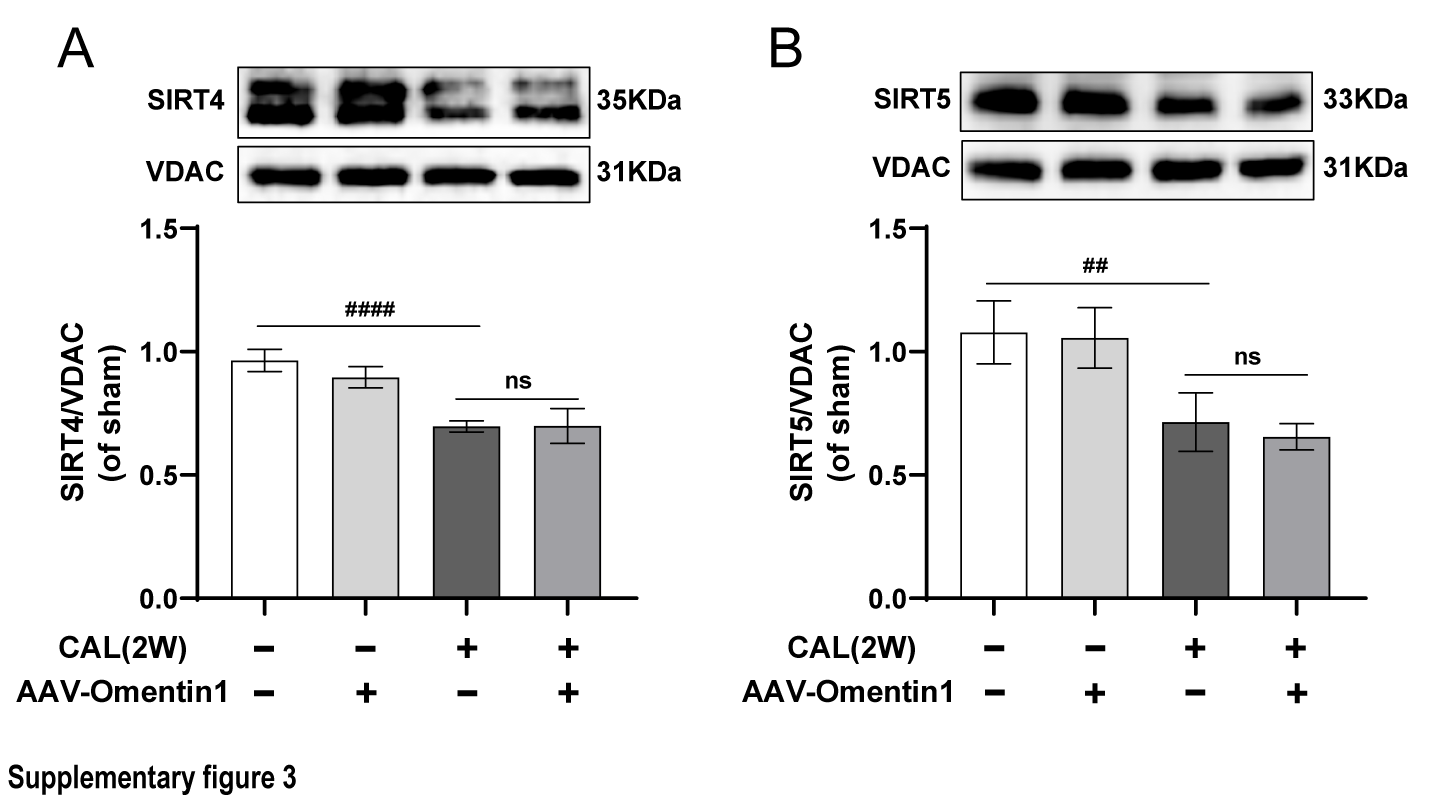


**Fig. S3** Representative western blotting images of the expression of **A** SIRT4 and **B** SIRT5 in myocardial mitochondria of mice with fat-specific omentin1 overexpression, and the statistical results were presented (n = 4). Results were gained from three independent experiments and were expressed as mean ± SD. ***^##^****P* < 0.01, ***^####^****P* < 0.0001 vs. the sham group. **A-B** one-way ANOVA.


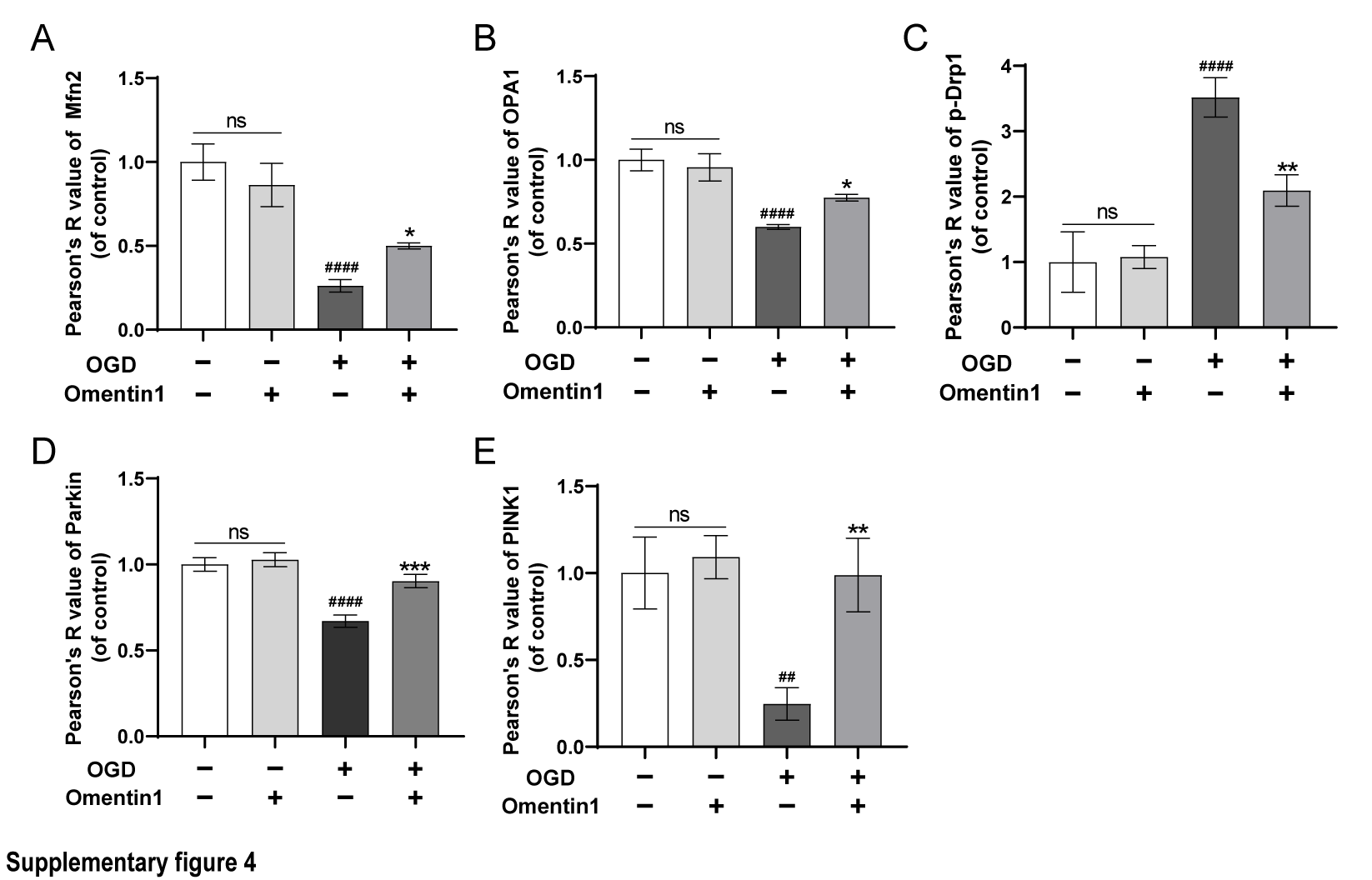


**Fig. S4** The statistical results of immunofluorescence analysis of Figure 5. Pearson’s correlation coefficient represents the degree of colocalization of **A** Mfn2, **B** OPA1, **C** p-Drp1(Ser616), **D** Parkin and **E** PINK1 with mitochondria (n = 3). Results were gained from three independent experiments and were expressed as mean ± SD. ***^##^****P* < 0.01, ***^####^****P* < 0.0001 vs. the control group; **P* < 0.05, ***P* < 0.01, ****P* < 0.001 vs. the OGD group. **A-E** one-way ANOVA.


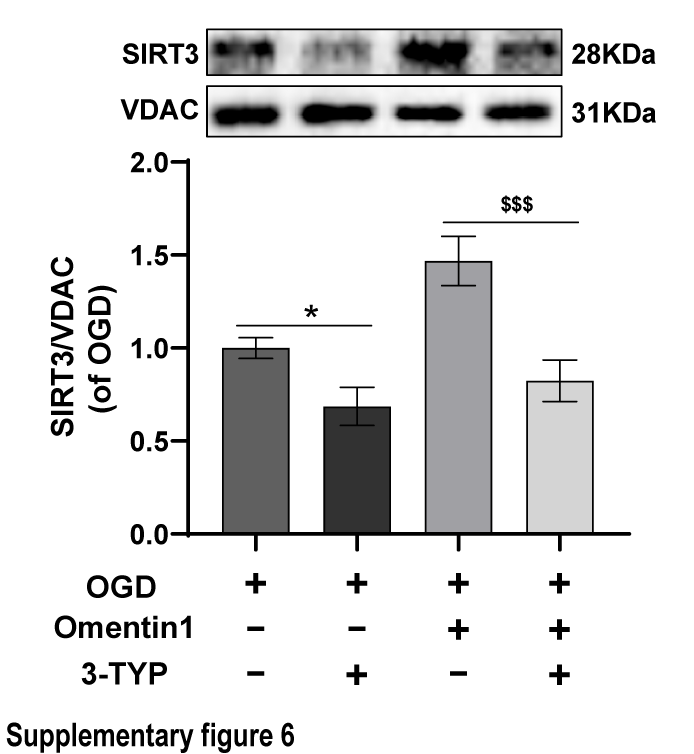


**Fig. S5** Representative images of western blotting analysis of the SIRT3 expression in mitochondria of cardiomyocytes treated with omentin1 and 3-TYP and the statistical result was presented (n = 3). Results were gained from three independent experiments and were expressed as mean ± SD. **P <* 0.05 vs. the OGD group; *^$$$^P <* 0.001 vs. the OGD + omentin1 group. One-way ANOVA.

**Supplementary Tables**

**Table S1.** The clinical characteristics of heart failure (HF) patients and healthy subjects

| Clinical Characteristics (n = 23/group) | HF patients | healthy subjects |
| --- | --- | --- |
| Age, yrs | 70.57 ± 2.32 | 56.96 ± 1.12 |
| Male/female | 14/9 | 15/8 |
| Pulse rate, times/min | 77.57 ± 3.32 | 74.22 ± 2.13 |
| Respiratory rate, times/min | 18.96 ± 0.28 | 18.78 ± 0.25 |
| Systolic blood pressure, mm Hg | 123.48 ± 3.24 | 120.48 ± 3.37 |
| Diastolic blood pressure, mm Hg | 74.00 ± 2.26 | 70.48 ± 1.94 |
| Glucose, mmol/L | 6.10 ± 0.48 | 5.58 ± 0.26 |
| Total cholesterol, mmol/L | 4.19 ± 0.25 | 4.06 ± 0.23 |
| Triglyceride, mmol/L | 1.50 ± 0.25 | 0.98 ± 0.08 |
| HDL cholesterol, mmol/L | 1.15 ± 0.04 | 1.65 ± 0.08 |
| LDL cholesterol, mmol/L | 4.23 ± 0.19 | 2.82 ± 0.09 |
| CRP, mg/L | 10.78 ± 2.31 | 6.35 ± 0.48 |
| CK-MB, U/L | 18.91 ± 2.29 | 10.22 ± 0.73 |
| LDH, U/L | 317.91 ± 39.27 | 193.17 ± 9.57 |
| cTnI, ug/L | 0.21 ± 0.03 | 0.10 ± 0.02 |
| BNP, pg/ml | 970.39 ± 180.76 | 64.30 ± 3.54 |
| Omentin, ng/mL | 91.05 ± 6.91 | 157.03 ± 7.19 |

Values were presented as the mean ± SEM. LDL = low-density lipoprotein; HDL = high-density lipoprotein; CRP = C-reactive protein; CK-MB = creatine kinase MB isoenzyme; LDH = lactate dehydrogenase; cTnI = cardiac troponin I; BNP = brain natriuretic peptide.
